# Socio-Environmental Factors Associated with the Risk of Contracting Buruli Ulcer in Tiassalé, South Côte d’Ivoire: A Case-Control Study

**DOI:** 10.1371/journal.pntd.0004327

**Published:** 2016-01-08

**Authors:** Raymond T. A. S. N’krumah, Brama Koné, Issaka Tiembre, Guéladio Cissé, Gerd Pluschke, Marcel Tanner, Jürg Utzinger

**Affiliations:** 1 Département Recherche et Développement, Centre Suisse de Recherches Scientifiques en Côte d’Ivoire, Abidjan, Côte d’Ivoire; 2 Unité de Formation et de Recherche des Sciences Médicales, Université Félix Houphouët-Boigny, Abidjan, Côte d’Ivoire; 3 Institut de Gestion Agropastorale, Université Péléforo Gon Coulibaly, Korhogo, Côte d’Ivoire; 4 Swiss Tropical and Public Health Institute, Basel, Switzerland; 5 University of Basel, Basel, Switzerland; Fondation Raoul Follereau, FRANCE

## Abstract

**Background:**

Buruli ulcer (BU) is a cutaneous infectious disease caused by *Mycobacterium ulcerans*. The exact mode of transmission remains elusive; yet, some studies identified environmental, socio-sanitary, and behavioral risk factors. The purpose of this study was to assess the association of such factors to contracting BU in Tiassalé, south Côte d’Ivoire.

**Methodology:**

A case-control study was conducted in 2012. Cases were BU patients diagnosed according to clinical definition put forth by the World Health Organization, readily confirmed by IS2404 polymerase chain reaction (PCR) analysis prior to our study and recruited at one of the health centers of the district. Two controls were matched for each control, by age group (to the nearest 5 years), sex, and living community. Participants were interviewed after providing oral witnessed consent, assessing behavioral, environmental, and socio-sanitary factors.

**Principal Findings:**

A total of 51 incident and prevalent cases and 102 controls were enrolled. Sex ratio (male:female) was 0.9. Median age was 25 years (range: 5–70 years). Regular contact with unprotected surface water (adjusted odds ratio (aOR) = 6.5; 95% confidence interval (CI) = 2.1–19.7) and absence of protective equipment during agricultural activities (aOR = 18.5, 95% CI = 5.2–66.7) were identified as the main factors associated with the risk of contracting BU. Etiologic fractions among exposed to both factors were 84.9% and 94.6%, respectively. Good knowledge about the risks that may result in BU (aOR = 0.3, 95% CI = 0.1–0.8) and perception about the disease causes (aOR = 0.1, 95% CI = 0.02–0.3) showed protection against BU with a respective preventive fraction of 70% and 90%.

**Conclusions/Significance:**

Main risk factors identified in this study were the contact with unprotected water bodies through daily activities and the absence of protective equipment during agricultural activities. An effective strategy to reduce the incidence of BU should involve compliance with protective equipment during agricultural activities and avoidance of contact with surface water and community capacity building through training and sensitization.

## Author Summary

In Côte d’Ivoire, West Africa, Buruli ulcer (BU) is the second leading cause of mycobacterial infection after tuberculosis. Moreover, Côte d’Ivoire is one of the most affected countries worldwide by BU. Studies suggest that the reservoir of this mycobacterial infection is environmental, but the exact mode of disease transmission is still not known. The main strategy for the control of BU is early detection of cases and to treat them with antibiotics. In order to improve our understanding of the risk for contracting BU, which would allow primary prevention of the disease, a case-control study was conducted in 2012 in the district of Tiassalé, located in the southern part of Côte d’Ivoire, where the rain forest meets the savannah. We found that regular contact with unprotected surface water was an important factor associated with the risk of contracting BU. Such water contacts occur during agricultural activities, as well as washing, bathing, and swimming practices. An effective strategy to prevent BU in the district of Tiassalé–and perhaps in other endemic areas–will have to focus on improved access to clean water at the household, and sensitization and education for better practices in subsistence agriculture.

## Introduction

Buruli ulcer (BU) is an infectious skin disease caused by the environmental mycobacterium, *Mycobacterium ulcerans*. It is the third most common mycobacterial disease in the world in immunocompetent individuals, after tuberculosis and leprosy [1–3] and the second one in Côte d’Ivoire after tuberculosis [4]. It occurs at any age but the majority of sufferers are children aged below 15 years [5–7], mainly in tropical and subtropical regions, in rural marshy areas [8–11]. According to the World Health Organization (WHO), the countries most affected by BU in the past 10 years were Benin, Côte d’Ivoire, and Ghana in Africa; French Guyana in Latin America; and Australia and Papua New Guinea in Oceania [12]. The reservoir of *M*. *ulcerans* seems to be environmental, but the exact mode(s) of disease transmission remain to be elucidated [13,14].

There are multiple endemic foci of BU in Côte d’Ivoire, distributed throughout the country. The total number of cases reported between 2004 and 2014 was 19,145 [12]. In 1997, during the first national survey, 10,382 cases were identified with a mean national prevalence of 0.32 per 1,000 inhabitants [4]. Data suggest that national strategies for the management of BU do not reach the most heavily affected rural communities. Areas around cities such as Bouaké in central Côte d’Ivoire, Daloa in the west-central part of the country, and Tiassalé in the southern part are particularly affected. In 1998, a high prevalence of BU (up to 22%) was found in some villages of Bouaké and Daloa [4] and from 2004 to 2007, 112 cases of BU were recorded in a single village (Sokrogbo) of the district of Tiassalé [15].

Several epidemiologic studies have been conducted in Africa and some other parts of the world to identify risk factors for *M*. *ulcerans* disease. Frequently reported risk factors are the contact of individuals with stagnant or slow flowing surface water through swimming, fishing, laundry, washing dishes, water supply, etc. [16–20], wearing short clothes during agricultural activities [21,22], and agricultural land use [23]. Protecting factors have also been identified by those studies. BU risk reduction was associated with the use of protected water sources, hygiene practices such as the use of bath soap, alcohol use for wound care and for cleaning body areas concerned by insect bites, and wearing long clothing (long-sleeved shirts and pants, boots, and gloves) for agricultural activities [24,25].

In Côte d’Ivoire, very few studies [5,8] on risk factors associated to BU have been conducted. Ahoua et al. [5] showed in 2009 in the regions of Bouaké, Man, and Daloa that the occurrence of BU was significantly associated with the use of unprotected water (ponds, creeks, rivers and dams) and living or practicing agricultural activities nearby aquatic ecosystems. Earlier in 1995, Marston et al. [8] revealed the association of BU to agricultural activities in the region of Daloa. Wearing long pants was a protecting factor. In addition to the aforementioned risk factors, the role of insect bites in the transmission of *M*. *ulcerans* has been extensively studied worldwide and in Africa [11,15,17,21,26,27]. In Côte d’Ivoire, for example, Doannio et al. [15] revealed the molecular signatures of *M*. *ulcerans* in the tissues of two water bugs (genera *Micronecta* and *Diplonychus*) identified as potential vectors in the transmission of *M*. *ulcerans* in the village of Sokrogbo (Tiassalé district). In Benin, from a 1-year mosquitoes and aquatic insects sampling and analysis by qPCR, *M*. *ulcerans* was detected in around 8.7% of aquatic insects but never in mosquitoes (larvae or adults) or in other flying insects [27], although previous studies [17,21,26] indicated a role of mosquitoes as vectors in the transmission of BU. Another 1-year longitudinal sampling and analysis of aquatic macro-invertebrates and vertebrates in Cameroon [11] showed the presence of *M*. *ulcerans* in nearly all taxonomic groups of the aquatic community and it was approximately evenly distributed among the whole community.

To date, the precise mode of transmission of BU remains elusive. It has been assumed that the pathogen is transmitted to humans by direct contact of injured skin with contaminated water or through biting of some aquatic insects. Against this background, identification of risk factors associated with the onset of BU are crucial for prevention and control of BU. Those risk factors may vary from one region to another based on environmental, socio-economic, and behavioral patterns.

The region of Tiassalé is located in the forest-savannah transition zone in southern Côte d’Ivoire, crossed from North to South by two main rivers (i.e., Bandama and N’zi). The region, which, before 1980, was ranked among the minor zones of BU, gradually became an important focus of BU shortly after the construction of a hydroelectric dam in the late 1970s. Indeed, Tiassalé became a wetland, characterized by subsistence farming, including lowland rice cultivation and swamps. Generally speaking, there are only few epidemiologic studies pertaining to BU in Côte d’Ivoire [5,8]. In the region of Tiassalé, apart from one study on the potential vectors of BU [15], no investigations on risk factors were carried out. The ecologic or environmental characteristics (wet, marshy areas) and behavioral patterns (agriculture, water access for household) of that region might explain the high endemicity of BU.

The main goal of the current study was to deepen our understanding of the transmission process of BU in Tiassalé for an efficient control of the disease. Specific objectives were (i) to assess risk factors of BU in the district of Tiassalé; (2) to determine etiologic and preventive fractions among exposed groups; and (3) to suggest efficient control strategies.

## Methods

### Ethics Statement

This research was carried out in the frame of a project entitled “Ecohealth approach in water and health management under climate change: adaptation strategies to drought and flooding events in four countries in West Africa”, implemented from 2009 to 2013. In Côte d’Ivoire, the National Ethics Committee cleared the research protocol (reference no. 5383/MSHP, dated 28 October 2009). In addition, agreement and a letter of support were obtained from the district medical officer at Tiassalé to collect data in the health centers of the district. Due to high illiteracy rates among rural dwellers in Tiassalé, oral informed consent was obtained from adults (cases and controls) or from parents (or legal guardians) of any individual aged below 18 years (cases and controls), in the presence of an eye witness (health worker) before enrolment and interview. The ethics committee explicitly approved this consent procedure. Participation was voluntary, and hence, people could withdraw anytime without further obligation. In Côte d’Ivoire, the treatment of BU is free of charge according to national guidelines [28,29] and cases were recruited and interviewed when they came for their daily free treatments.

### Study Design and Case Definition

A case-control study was conducted in the district of Tiassalé. Incident and prevalent cases were recruited between18 August and 10 September 2012 at the hospital and rural health centers of the district while they were seeking care.

Cases were defined as any BU patient diagnosed according to WHO clinical definition [14,30] and confirmed by IS2404 polymerase chain reaction (PCR) analysis conducted at the Institut Pasteur in Abidjan. Patients of all ages and both sex were enrolled, most of whom lived in the district of Tiassalé and presented at the local health centers or the hospital.

Controls were defined as patients with diseases other than BU who were seeking care at the same hospital or health centers as the cases. Controls were randomly selected and matched to cases to the nearest 5 years, sex, and type of residency (rural or urban). Two controls were selected for each case.

### Study Area

**Fig 1:**
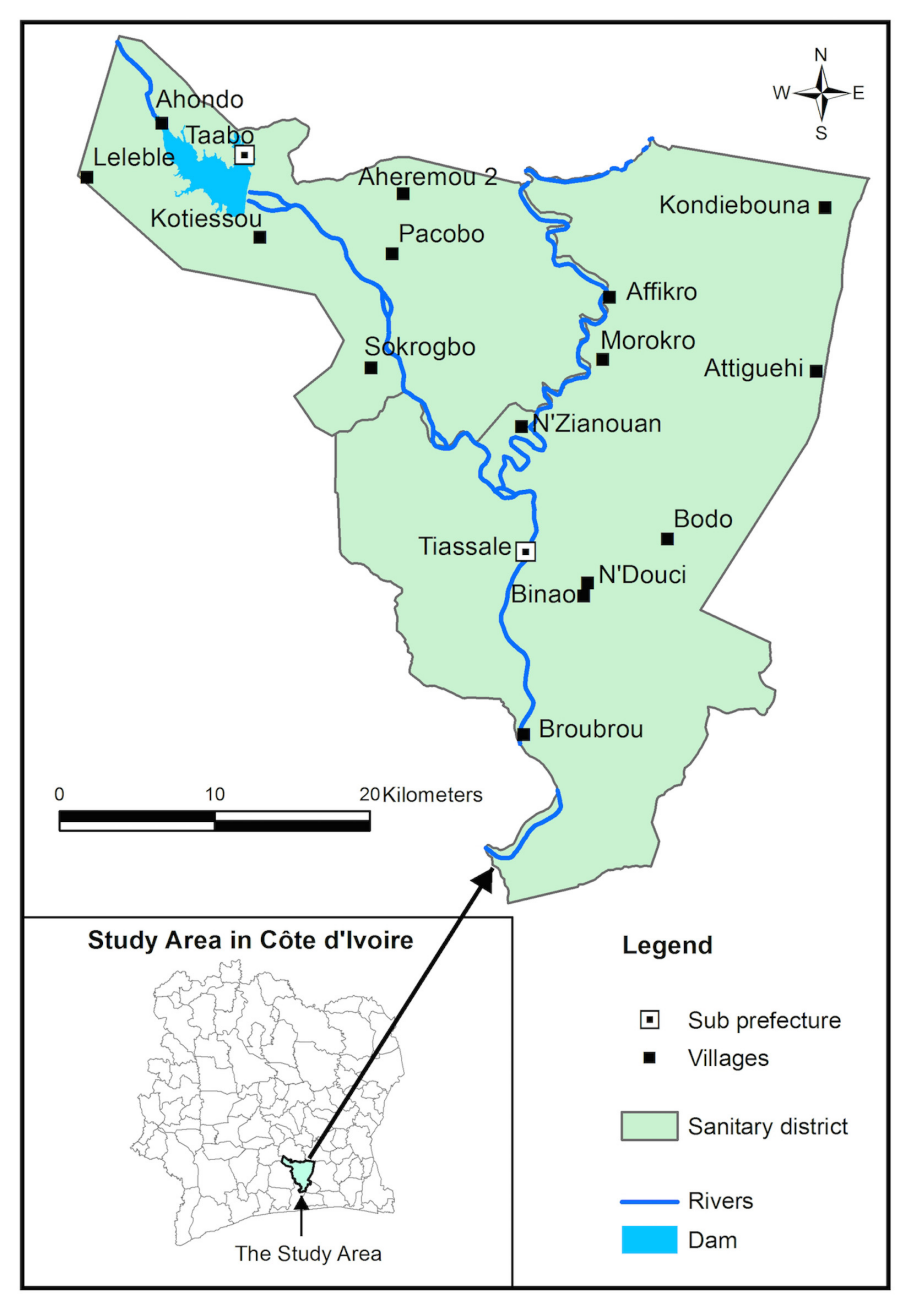
Map of the district of Tiassalé, in south Côte d’Ivoire.

The district of Tiassalé is located in southern Côte d’Ivoire, extending from latitude 5°32' N to 6°24' N, and from longitude 4°29' W to 5°14' W. The district is composed of two sub-prefectures: Tiassalé and Taabo. In Taabo, a hydroelectric dam was constructed across the Bandama River in the late 1970s that formed an impoundment of approximately 69 km^2^ [31–33] (Fig 1).

According to the General Census of Population and Housing of 2014, the total population of the district of Tiassalé was 263,495 [34]. The climate is tropical and humid. The average annual rainfall is 1,740 mm with an average annual temperature of 26.6°C [35,36]. The population of the Tiassalé district is predominantly rural and people are mainly engaged in subsistence farming.

### Sample Size

The size of the sample was estimated using EpiInfo (version 3.5.3) sample calculation tool. We considered an unprotected surface water exposure frequency of 25% [5,8]; set alpha to 5%, and a power to 80% [22]. Our aim was to identify risk factors with an odds ratio (OR) of 3 or higher. Hence, a minimum of 50 cases and 100 controls were required.

### Data Collection

Cases were recruited at the hospital of the city of Tiassalé and at the health centers of the villages of Taabo, Ahondo, Kotiéssou, Léléblé, N’Doucy, N’Zianouan, and Sokrogbo, all located in the district of Tiassalé. At the hospital of Tiassalé, the research team met the district medical officer who introduced them to the coordinator of BU control in the district, also responsible of the treatment of cases at the hospital, and to nurses in charge of BU treatment in the villages. In each health center, the research team came in the morning (from 7 a.m. to noon), stayed with the BU treatment team and recruited within persons coming for their daily treatment. For each case, two controls were recruited in the same center at the reception desk, by a two-stage sampling method [5,37,38]. Having explained the purpose of the study to case-matched patients waiting for consultation at the desk, numbers were associated to each patient having accepted to participate in the study. The two controls were chosen at random within the patients with numbers. When only two case-matched patients were available at the moment of selection, they were selected. In case of absence of matching patients, they were recruited when new patients came or the following days. Patients were interviewed after their consultation and care.

After having obtained oral informed witnessed consent, with the BU care givers as witness, a pre-tested questionnaire was administered to cases and controls to collect information on their socio-demographic status (e.g., age, sex, education, and marital status) and their knowledge, attitude, and practices (e.g., agricultural practices, wearing protective clothing during such activities, swimming, fishing, living and working place, source of drinking water, vaccination against Bacilli Calmette-Guérin (BCG), etc.). BCG vaccination was assessed by identifying the presence of the scar on the left shoulder around the deltoid region. Clinical features of cases were also assessed, including clinical forms and categories, location of lesions, signs, and symptoms.

A questionnaire was administrated by trained field enumerators either in French or translated to one of the local languages (i.e., Baoulé and Malinké). If need be, a community health worker assisted the field enumerators during the interviews.

### Statistical Analysis

Data quality control was systematically conducted on all questionnaires. Data were processed and analyzed using EpiInfo, version 3.5.3 (Centers for Disease Control and Prevention; Atlanta, United States of America).

BU was the dependent variable, while socio-demographic factors, knowledge, attitude, and practices of participants, environmental patterns of their living and working places were independent variables. All variables were described through proportions. Univariate analysis was used to describe the association between BU and independent variables. Statistical significance was determined by consulting 95% confidence intervals (CIs), checking whether or not 1 was included for the observed ORs.

Multiple conditional logistic regression analysis using a step down backward elimination process was performed to identify the factors that are significantly associated with BU firstlyand secondly to control possible confounding factors. At each regression, factors associated to BU with a p-value of the Wald test (Z statistic) greater 0.25 were eliminated. We then removed, one by one, the explanatory variables with p-values between 0.05 and 0.25 until the final model with variables associated to BU with a p-value less than or equal to 0.05.

The etiological fraction among people exposed to risk factors (EFe) and the preventive fraction among people exposed to protective factors of BU (PFe) were estimated.

## Results

Overall, 51 cases and 102 controls were enrolled. Questionnaires were filled in during the survey, registered for data analysis, and quality-controlled before statistical analysis.

### Socio-Demographic Characteristics of Cases

Table 1 shows the main socio-demographic and clinical characteristics of cases. Among the 51 BU cases, 24 (47.1%) were males, thus the sex ratio (male/female) was 0.9. The age of the BU cases ranged between 5 and 70 years with a median age of 25 years. About half of the BU cases were in the age range of 15–35 years. Among adult BU cases, the main socioeconomic activity is subsistence farming. The proportion of agricultural activities among controls was significantly lower (45.1% *versus* 28.4%; p<0.05). Among the 51 BU cases interviewed, 29 (56.9%) had no formal school education. The respective proportion among controls (39.2%) was significantly lower (p<0.05).

**Table 1:** Socio-demographic and clinical characteristics of BU cases in the district of Tiassalé, south Côte d'Ivoire (community-matched case control study, August-September 2012).

Characteristics	BU cases (n, %)	Characteristics	BU cases (n, %)
N	51 (100)	Type of BU cases	
**Sex**		Incident cases	2 (4.0)
Male	24 (47.1)	Prevalent cases	49 (96.0)
Female	27 (52.9)		
		BCG* **vaccination status**	
**Age in years (median, range)**	25 (5–70)	Vaccinated	33 (64.7)
˂10	4 (7.8)	Unvaccinated	18 (35.3)
10–14	9 (17.6)		
15–25	16 (31.4)	**Early symptoms**	
26–35	9 (17.6)	Small button on the skin	44 (86.3)
˃35	13 (25.5)	Swelling of the body part	7 (13.7)
**Professional activities**		**Clinical forms**	
Agriculture	23 (45.1)	Nodule/plaque	0 (0.0)
Student	9 (17.6)	Oedema	2 (4.0)
Official	1 (2.0)	Ulceration	49 (96.0)
No activity	13 (25.5)		
Other	5 (9.8)	**Localisation of lesion**	
		Lower limbs	39 (76.5)
**Educational attainment**		Upper limbs	9 (17.6)
Primary	14 (27.5)	Other parts	3 (5.9)
Secondary	8 (15.7)		
No education	29 (56.9)	**Lesions classification**	
		Category I (D** ˂5 Cm)	2 (3.9)
**Marital status**		Category II (D: 5–15 Cm)	26 (51.0)
Married/cohabiting	22 (43.1)	Category III (D ˃ 15 Cm)	23 (45.1)
Single/widow(er)	29 (56.9)		

*: Bacille Calmette-Guérin

**: Diameter

### Clinical Features and Management of BU Patients

**Fig 2:**
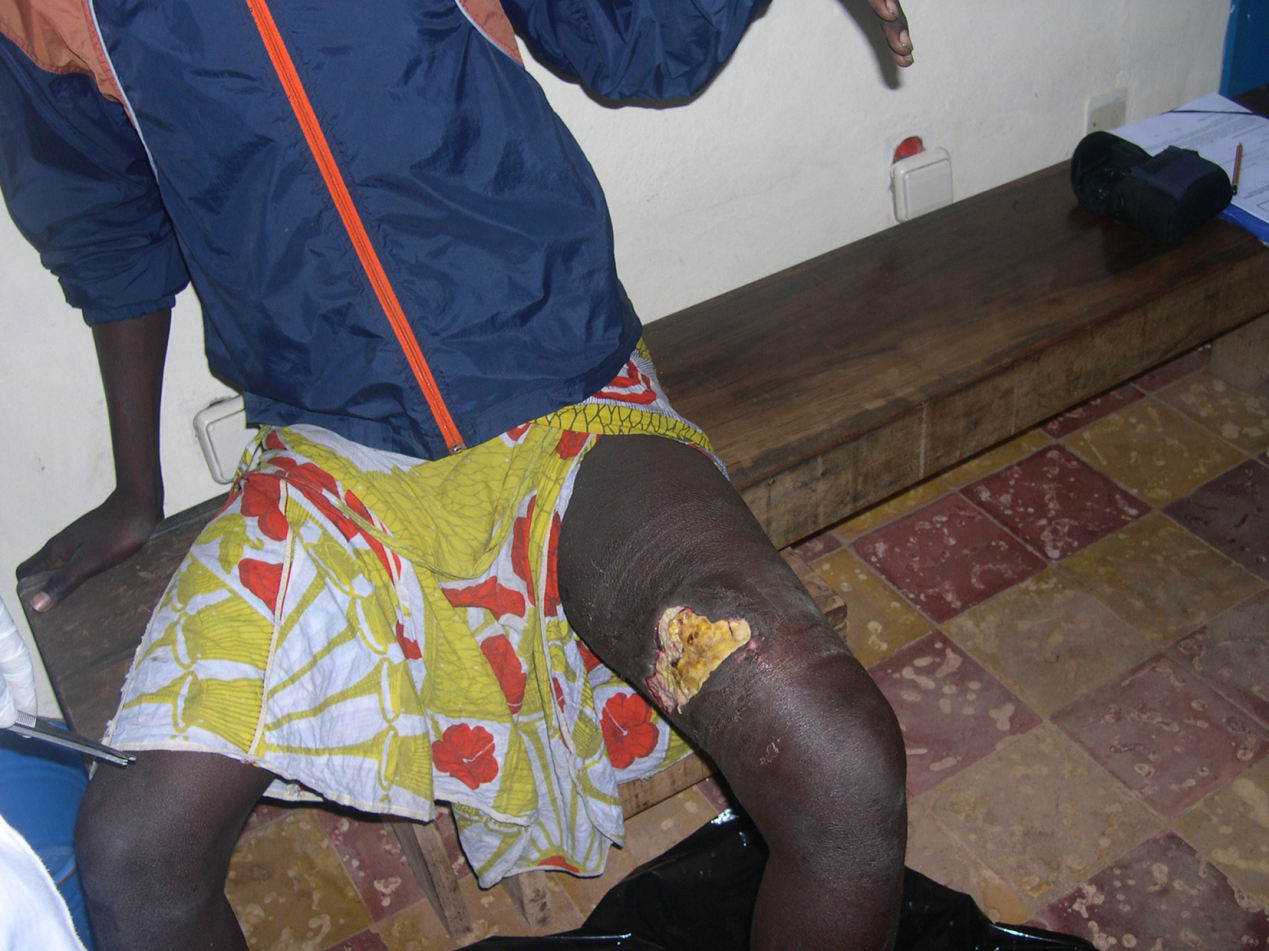
Buruli ulcer case in the sanitary district of Tiassalé, south Côte d’Ivoire (case-control study, August-September 2012, Source: N’krumah et al.).

Most cases of BU recruited (96.0%) were prevalent cases (Table 1). We found that 64.7% of cases were vaccinated against BCG. The first symptom of BU in most of cases interviewed (86.3%) was the appearance of a small hard nodule on the skin. Oedematous forms, as another early sign of BU, were cited by another 13.7% of the cases. The main observed lesions at time of data collection were ulcers (96.1%), most frequently located on the lower limbs (76.5%) or upper limbs (17.5%) with a predominance of lesions of category II (51.0%), characterized by a lesion diameter of 5–15 cm (Fig 2).

Most of BU patients (84.3%) reported that their care through the existing health system in the district of Tiassalé was provided free of charge, while 15.7% of cases reported specific costs incurred for medical bandages and compresses. The majority of cases (90.0%) used traditional medicine as the first means of care. Two-third (36/51; 66.6%) of BU cases came at a health center for their first consultation more than 2 months after the onset of the disease; 29.4% between 1 and 2 months, and only 5.8% reached a health center less than 1 month after disease onset.

### Univariate Analysis of Factors Associated with the Risk of Contracting BU

#### Socio-demographic factors associated with contracting BU

Illiteracy was positively associated with contracting BU (OR = 2.0, 95% CI = 1.0–4.0) (Table 2). A lack of knowledge on the risks of contracting BU (OR = 3.0, 95% CI = 1.5–6.0) was significantly associated with its occurrence. The EFe was 66.4% (95% CI = 32.1–83.4%). Most subjects (59.5%) who lacked good knowledge about risk factors for contracting BU attributed it to a mystical cause. The EFe was 63.0% (95% CI = 22.4–82.3%).

**Table 2:** Univariate analysis of selected variables for BU in the district of Tiassalé, south Côte d'Ivoire (case-control study, August-September 2012).

	No. (%) of cases	No. (%) of controls	Univariate OR	EFe or PFe
Characteristics	Subject (n = 51)	Subject (n = 102)	(95% CI)	(95% CI
**Socio-demographic factors**				
Education status: no education/primary and secondary	29 (56.9)	40 (39.2)	2.0 (1.0–4,0)	51.1 (3.2–75.3)
Knowledge about the risk which may result in BU: little/good	34 (66.7)	41 (40.2)	3.0 (1.5–6.0)*	66.4 (32.1–83.4)
Perception about BU: mystical disease/natural disease	38 (74.5)	53 (52.0)	2.7 (1.3–5.7)*	63.0 (22.4–82.3)
**Environmental factors and water contact activities**				
Regular contact with a surface water source (river/creek/dam/lake): yes/no	36 (70.6)	21 (20.6)	9.3 (4.3–20.0)*	89.2 (76.7–95.0)
Distance between a surface water point and place of residence: ˂500 m/˃500 m	30 (58.8)	39 (38.2)	2.3 (1.2–4.6)*	56.7 (14.0–78.2)
Regular agricultural activities (irrigated rice): yes/no	40 (78.4)	40 (39.2)	5.6 (2.6–12.3)*	82.3 (61.4–91.8)
Regular washing/bathing/swimming in surface water: yes/no	41 (80.4)	63 (61.8)	2.5 (1.1–5.6)*	60.6 (12.5–82.3)
Regular water supply at a surface water point (river/creek/dam): yes/no	34 (66.7)	39 (38.2)	3.2 (1.6–6.6)*	69.0 (37.3–84.7)
Wearing protective equipment (boots/gloves/long pants and clothes) during agricultural activities: no/yes	22 (43.1)	74 (72.5)	0.3 (0.1–0.6)*	71.3 (41.9–85.8)
**Insect bites**				
Insect bites during water contact activities: yes/no	32 (62.7)	40 (39.2)	2.6 (1.3–5.2)	61.7 (23.4–80.8)
**Health-related factors**				
Presence of local trauma on the skin during water contact activities: yes/no	27 (52.9)	47 (46.1)	1.3 (0.7–2.6)	24.0 (1.0 61.3)
BCG vaccination status: unvaccinated/vaccinated	18 (35.3)	62 (60.8)	0.4 (0.2–0.7)*	64.8 (29.2–82.5)

* Significant association between variable and BU

CI, confidence interval; EFe, etiologic fraction among the exposed; PFe, preventive fraction among the exposed; OR, odds ratio

#### Environmental and behavioral factors associated with contracting BU

The regular contact with open surface water (i.e., river, pond, creek, and dam) was associated with higher odds of contracting BU (OR = 9.3, 95% CI = 4.3–20.0). The EFe due to regular contact with these open sources was 89.2% (95% CI = 76.7–95.0%). Proximity of households to water points was positively associated with the occurrence of BU (OR = 2.3, 95% CI = 1.2–4.6). The EFe was 56.7% (95% CI = 14.0–78.2%).

The most risky daily activities directly or indirectly related to water contact were farming (rice and vegetables) and fishing (OR = 5.6, 95% CI = 2.6–12.3), contacting water for household supply at surface water points (OR = 3.3, 95% CI = 1.6–6.6) (S1 Fig), and washing and/or bathing at surface water points (OR = 2.5, 95% CI = 1.1–5.6). The EFe due to agricultural activities (farming/fishing) was 82.3% (95% CI = 61.4–91.8%). Those of water supply and laundry activities/swimming at water points were respectively 69.0% (95% CI = 37.3–84.7%) and 60.6% (95% CI = 12.5–82.3%).

The regular wearing of protective equipment (OR = 0.3, 95% CI = 0.1–0.6), such as boots, gloves, and long clothing that cover large parts of the body was associated with low odds of contracting BU. The PFe was 71.3% (95% CI = 41.9–85.8%).

#### Insect bites

The risk of contracting BU was significantly associated with insect bites (OR = 2.6, 95% CI = 1.2–5.1). The EFe was 61.7.9% (95% CI = 23.4–80.8%).

#### Health-related factors

A history of skin trauma was not significantly associated with the occurrence of BU (OR = 1.3, 95% CI = 0.7–2.6). A history of BCG vaccination (OR = 0.4, 95% CI = 0.2–0.7) was associated with low odds of contracting BU. The PFe was 64.8% (95% CI = 29.2–82.5%).

### Multivariate Analysis of Factors Associated with the Risk of Contracting BU

Regular contact with unprotected water points (i.e., pond, creek, river, and dam) was associated with the occurrence of BU (adjusted OR (aOR) = 6.5, 95% CI = 2.1–19.7) with an etiologic fraction (EFe) of 84.9% (Table 3). This contact with the water points is made through agriculture activities and fishing (aOR = 6.3, 95% CI = 1.8–21.9) (S2 Fig) and washing/bathing/swimming activities (aOR = 7.5, 95% CI = 2.0–27.8) (S3 Fig) with respective EFe of 84.1% and 86.7%. Also the absence of protective equipment (e.g., boots, gloves, long sleeved shirts, and pants) for agricultural activities or in contact with surface water was associated with a higher risk of contracting BU (aOR = 18.5, 95% CI = 5.2–66.7) and an EFe of 94.6%. A good knowledge of people on the risk factors of BU (aOR = 0.3, 95% CI = 0.1–0.8) and a good perception about BU causes (aOR = 0.1, 95% CI = 0.02–0.3) were protective against the disease with respective preventive fractions (PFe) of 70% and 90%.

**Table 3:** Multivariate backward elimination model of conditional logistic regression for risk factors for BU in the district of Tiassalé, south Côte d'Ivoire (case-control study, August-September 2012).

Characteristics	aOR (95% CI)	Coef	EFe or PFe (%)	P value
Regular contact with a surface point	6.5 (2.1–19.7)	1.9	84.9	0.001*
Agricultural activities in contact with surface water	6.3 (1.8–21.9)	1.8	84.1	0.004*
Absence of protective equipment during agricultural activities in contact with surface water	18.5 (5.2–66.7)	2.9	94.6	˂0.001*
Washing/bathing/swimming in a surface water	7.5 (2.0–27.8)	2.0	86.7	0.003*
Good knowledge about the risks that may result in BU	0.3 (0.1–0.8)	-1.4	70.0	0.021*
Good perception about the disease causes	0.1 (0.02–0.3)	-2.4	90.0	˂0.001*
CONSTANT*		-4.3	*	<0.001

* Statistically significant

aOR, adjusted odds ratio; CI, confidence interval; Coef. Coefficient; EFe, etiologic fraction among the exposed PFe:preventive fraction among the exposed

## Discussion

Our study identified significant risk factors associated with the occurrence of BU and estimated the impact of risk factors at the onset of this disease. We found that regular contact of people with unprotected water points, such as ponds, creeks, rivers, and dams was significantly associated with the occurrence of BU with about 85% of cases attributable to regular contact with unsafe surface water. This contact with water points was mainly due to agricultural activities (e.g., rice farming, market gardening, and fishing) and washing/bathing/swimming activities. In addition, a lack of knowledge on BU risk factors was significantly associated with the occurrence of the disease. In fact, it was observed that the majority of the participants associated BU to mystical causes. However, wearing protective equipment (e.g., boots, gloves, long sleeved shirts, and pants) before getting in contact with surface water was a protective factor against BU.

Case-control studies have several limitations, including the potential for recall bias that might have negatively affected our study. Most of the cases enrolled in our study were living with the disease more than one year and we assumed that the association of disease persistence may be confounded with disease development [22]. Two cases were 5-year-old and the others were aged 9 years and above. For the 5-year-old cases, parents/guardians answers may have been biased. Also, our study may suffer of a selection bias, as we recruited only cases coming to health centers for care, and hence, they have been included at different time points after receiving the diagnosis. They could have already received some basic information on the disease. This is likely to introduce a recall bias. We tried to minimize recall bias in the current study by applying a well-designed (mindful of cultural and context sensitivity) and thoroughly pre-tested questionnaire, followed by a careful multivariate analysis to separate risk factors and confounders. The minimum odds ratio (3.0) used for determining the size of the respondents could look high and may have make some associations too weak to observe.

However, the results found in our study are similar to several other epidemiologic studies conducted worldwide to identify risk factors associated with the occurrence of BU [5,8,17,20,22,24,25,39] validating in such our results. As also shown by our results, clinical diagnosis and management of BU in Africa mostly occurs at a late stage when the ulcer is already present and has a sizable dimension [4,40–43]. The Ivorian national BU survey carried out in 1997 found a proportion of ulcerated forms of 86.5% for reported cases [4]. This proportion is less than the one obtained in our study (96.1%). Both of the proportions may result from the late recourse to medical care by BU cases. The main socioeconomic activity of BU cases in the district of Tiassalé is subsistence agriculture (45.1%). Indeed, rice production is made around the dam and in humid lowlands. Berliat [40] found a similar proportion (47.0%) in a prior case-control study carried out in Manikro in central Côte d'Ivoire. A case-control study conducted in neighbouring Ghana showed that 44.0% of BU patients were engaged in subsistence agriculture [44].

In our study most patients with BU live in rural areas (78.4%) where access to clean water for family use is difficult, forcing the population to collect water from unprotected surface sources. This observation might explain why the EFe due to regular contact with open surface water, such as creeks, rivers, lakes, and dams is very high (84.9%). A history of local skin trauma (e.g., wounds and boils) was not associated with the occurrence of BU. This result is in agreement with other studies conducted in Côte d’Ivoire [45–47]. Unlike univariate analysis showed that the risk of BU was significantly associated with insect bites, the conditional logistic regression analysis rejected this risk factor. However, the association of insect bites with the occurrence of BU has been described by several authors [48–53]. In this context, it is important to note that the contact with water bodies that is strongly associated with the risk of contracting BU in our study may well be linked to aquatic insect bites that occur during such contact periods.

About half of the BU cases in our study were aged between 15 and 35 years. This observation is in contrast to prior studies observing that school-aged children are the most affected [4,6,38]. A possible explanation is that patients in the age group 15–35 years can travel more easily to hospitals or heath centers for care, as opposed to children below the age of 15 years who must be accompanied by parents/guardians who are busy with their day-to-day agricultural and other livelihood activities. Beyond the risk factors in the onset of BU that have been described, this study measured the share accountability of these risk factors in determining the EFe. This measure of the impact of the risk factors is missing in the majority of the studies on risk factors we reviewed.

To enhance the prevention and control of BU in the endemic health district of Tiassalé, capacity building (awareness and education) in rural communities, placing particular emphasis on the risk of contracting BU through contact with surface water, the necessity of wearing protecting equipment during such activities, and on correcting wrong perception about the disease should be envisaged. Additionally, measures for improved access to clean water should be strengthened. The effect of such measures could have an effect beyond BU, as shown in recent studies targeting on a host of other neglected tropical diseases [54–56].

### Conclusions

Our study showed that the main risk factors for BU in the region of Tiassalé in south Côte d’Ivoire is the contact with unprotected water bodies through daily activities such as agriculture (e.g., rice cultivation and fishing), laundry, and bathing. A good knowledge on the disease transmission process and the disease causes is likely to protect against it.

Compliance with protective equipment during agricultural activities and/or contact with surface water and community capacity building through training and sensitization on BU emerge as key strategies for the prevention of BU in the district of Tiassalé and potentially elsewhere in Côte d’Ivoire. Importantly, these measures could help avoid up to 95% of BU cases.

## Supporting Information

S1 Checklist STROBE checklist of items that should be included in reports of case-control studies. (PDF)

S1 Database Database of the study (case-control study, August-September 2012, Source: N’krumah et al.). (XLS)

S1 Fig A woman from Tiassalé district, collecting water at a surface water point (case-control study, August-September 2012, Source: N’krumah et al.). (TIF)

S2 Fig Rice cultivation site in the district of Tiassalé, South Côte d’Ivoire (case-control study, August-September 2012, Source: N’krumah et al.). (TIF)

S3 Fig Laundry and swimming activities in a village of Tiassalé district, South Côte d’Ivoire (case-control study, August-September 2012, Source: N’krumah et al.). (TIF)
