# Supplementary figures and images for: Socio-Environmental Factors Associated with the Risk of Contracting Buruli Ulcer in Tiassalé, South Côte d’Ivoire: A Case-Control Study
Source: PLoS Negl Trop Dis. 2016 Jan 8;10(1):e0004327. doi: 10.1371/journal.pntd.0004327 (PMC4712845; doi:10.1371/journal.pntd.0004327)

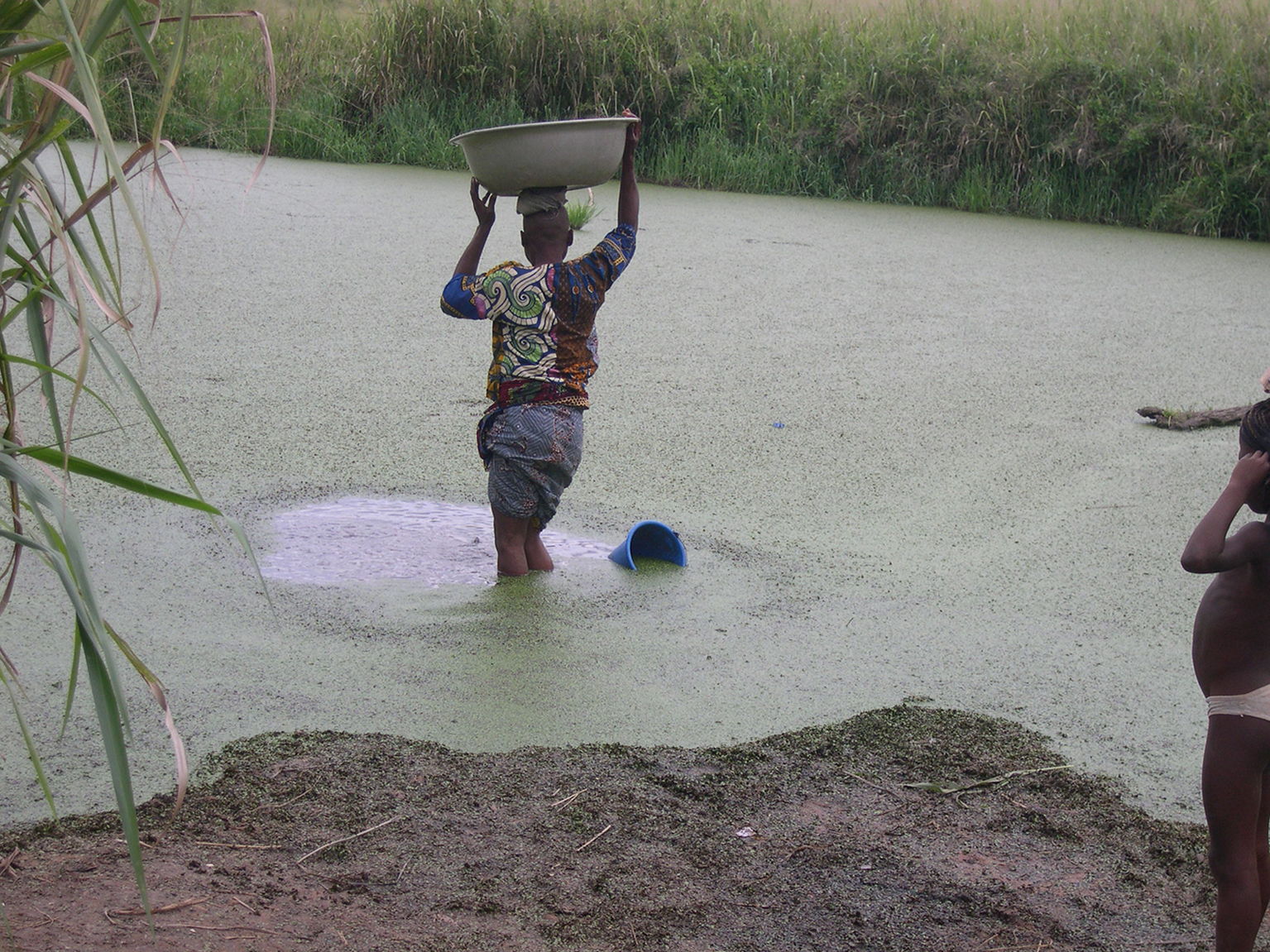

Supplement: S1 Fig — (TIF) [file pntd.0004327.s003.tif]

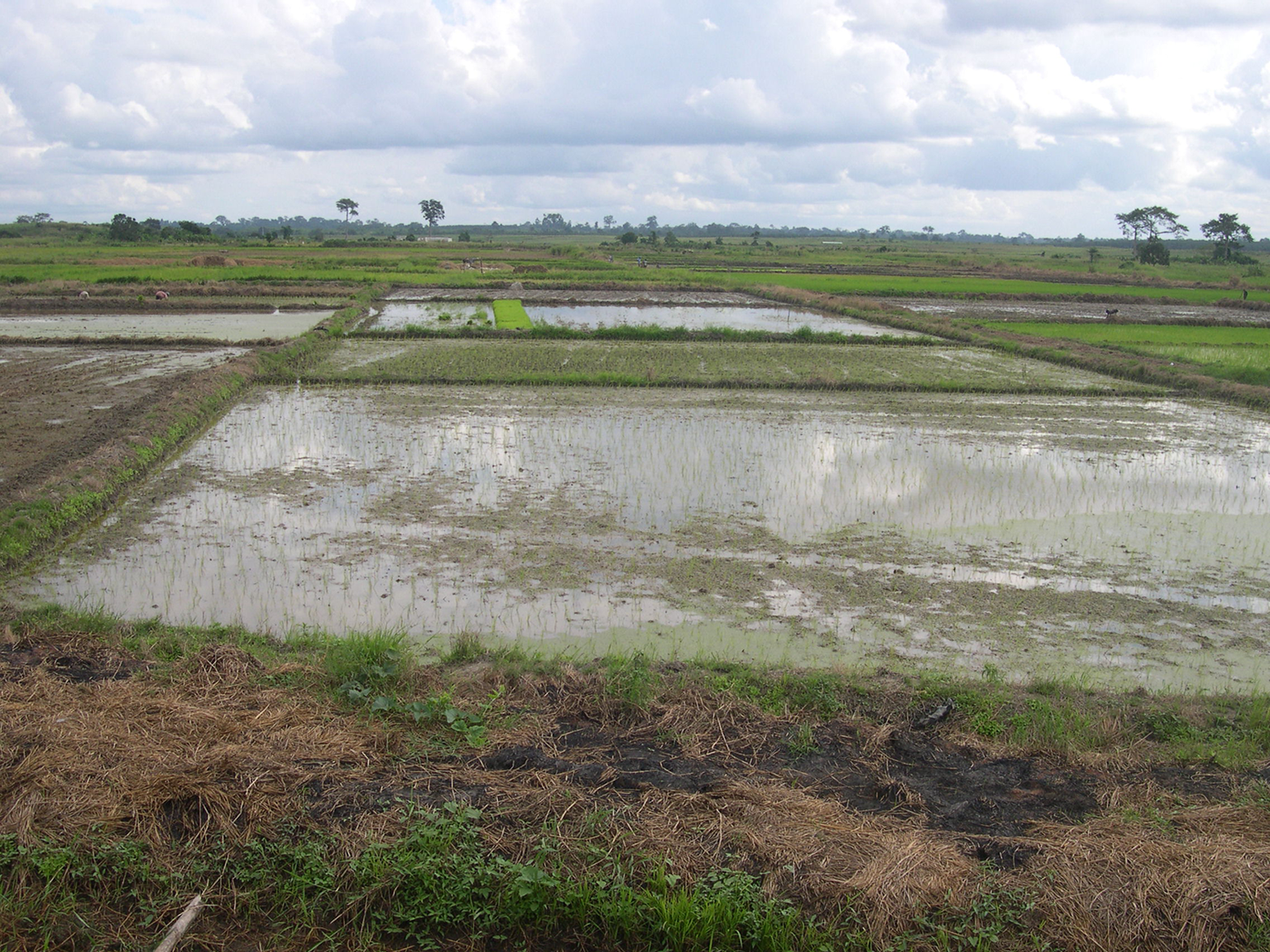

Supplement: S2 Fig — (TIF) [file pntd.0004327.s004.tif]

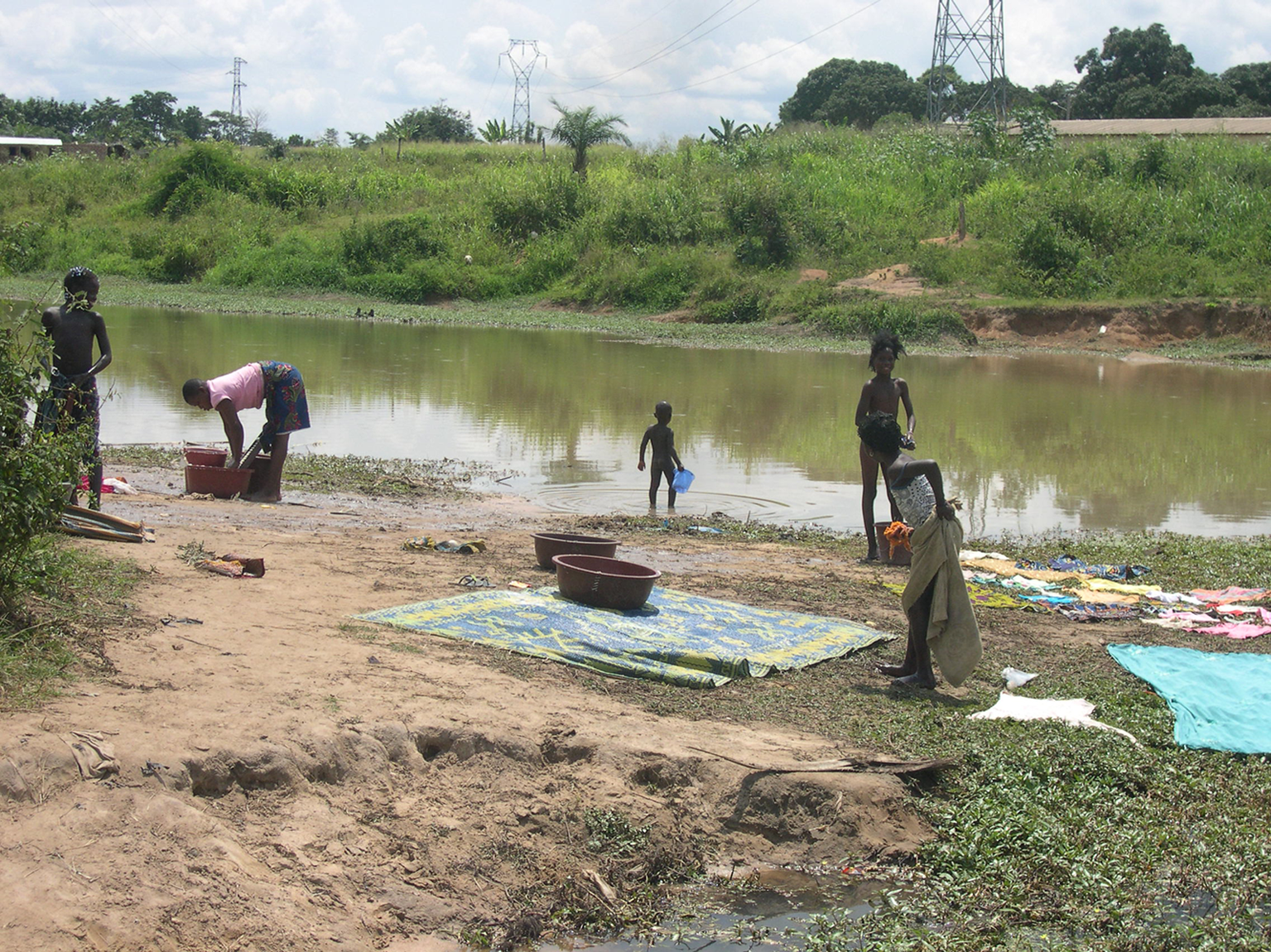

Supplement: S3 Fig — (TIF) [file pntd.0004327.s005.tif]
